# Proof of Concept: A Brief Psycho-Educational Training Program to Increase the Use of Positive Emotion Regulation Strategies in Individuals With Autism Spectrum Disorder

**DOI:** 10.3389/fpsyg.2021.705937

**Published:** 2021-11-01

**Authors:** Alexandra Zaharia, Katharina Noir-Kahlo, Nicolas Bressoud, David Sander, Daniel Dukes, Andrea C. Samson

**Affiliations:** ^1^Swiss Center for Affective Sciences, University of Geneva, Geneva, Switzerland; ^2^Faculty of Psychology, UniDistance Suisse, Brig, Switzerland; ^3^Institute of Special Education, University of Fribourg, Fribourg, Switzerland; ^4^Réseau Fribourgeois de Santé Mentale, Fribourg, Switzerland; ^5^Haute École Pédagogique du Valais, Saint-Maurice, Switzerland; ^6^Faculty of Psychology and Educational Sciences, University of Geneva, Geneva, Switzerland

**Keywords:** autism spectrum disorder, psycho-educational intervention, positive emotion regulation, emotion regulation strategies, behavioral intervention

## Abstract

Attenuated positive emotions and difficulties in regulating emotions are frequently observed in individuals with autism spectrum disorders (ASD) and are linked to increased risk of affective disorders, problematic behaviors, and impaired socio-emotional functioning. As such, interventions specifically focused on positive emotion regulation (ER) skills could be very valuable for individuals with ASD, their caregivers, and therapists. However, the field of positive ER in ASD is under-researched. The present study aimed at testing the practical potential and the preliminary effects of a brief novel psycho-educational training program on positive ER for individuals with ASD. Thirty male participants with ASD (aged 10–35years; *N*_training_=14, *N*_waitlist_=16) underwent a three-session program on the use of adaptive positive ER strategies (i.e., attentional deployment, cognitive change, and response modulation). Participants rated the program as easy to understand, interesting, pleasant, and likable. No dropouts or adverse effects were observed. The training group showed a significant increase in the self-reported use of the ER strategies compared to the waitlist group. The increase in the use of ER strategies maintained up to 7 weeks in the overall sample. Having reached high satisfaction rates and the intended effects in this proof of concept study, this novel program represents a promising tool to support ER. Future research should next investigate the efficacy of the intervention on day-to-day emotional experience and wellbeing.

**Clinical Trial Registration:** ClinicalTrials.gov # NCT02898298

## Introduction

Emotion-related difficulties, such as affective disorders (anxiety and depression) and problematic behaviors (tantrums and aggression), are highly prevalent in individuals with autism spectrum disorder (ASD) and persist across the life span (Gotham et al., 2015; Mikita et al., 2015; Patel et al., 2017; Hollocks et al., 2018; Hudson et al., 2019). These difficulties, identified as markers of emotion regulation impairments (Mazefsky et al., 2013; Richey et al., 2015; Berkovits et al., 2017), are linked to maladaptive behavior, negative mental health outcomes, and impaired socio-emotional functioning in individuals with ASD, affecting not only school inclusion and transition into professional life (Ashburner et al., 2010; Fage, 2015; Marsh et al., 2017) but also their own and their families’ quality of life (Mazefsky and White, 2014; Samson et al., 2014b; Hurd, 2017; Cai et al., 2018; Nuske et al., 2018). Emotion regulation (ER) can be defined as the attempt to influence which emotions one has, when one has them and how one experiences and expresses them (Gross, 1998). ER plays a key role in socio-emotional development, and the few interventions designed to strengthen this skill have been beneficial for individuals with ASD (Reyes et al., 2019; Sandbank et al., 2020). Inspired by an apparent increasing awareness among researchers and clinicians regarding the need to attenuate ER impairments in individuals with ASD and the few but beneficial interventions, this study describes the development and the first evidence of a training program to increase adaptive positive ER strategies, i.e., strategies that aim at upregulating positive emotions.

Research studies and interventions mainly focus on downregulating negative emotions and much less on upregulating positive emotions. However, researchers have begun to recognize the benefits of positive ER, such as alleviating the undesirable effects of negative emotions, broadening the repertoire of resources, promoting resilience, and contributing to wellbeing (Fredrickson, 2004; Gross et al., 2006; Tugade and Fredrickson, 2007; Livingstone and Srivastava, 2012; Carl et al., 2013; Hechtman et al., 2013). Although a nascent field, several positive ER interventions have shown favorable outcomes in clinical (depression and anxiety) and non-clinical populations (Hechtman et al., 2013; Weytens et al., 2014; Fussner et al., 2015; Quoidbach et al., 2015; Taylor et al., 2016). Certain ER strategies have been found to support positive ER (Langston, 1994; Bryant, 2003; Gable et al., 2004; Quoidbach et al., 2010, 2015). First, attentional deployment strategies (e.g., immersion in the present moment, savoring, and vivid visualization of pleasant activities) seem to be efficient in increasing positive emotions and life satisfaction. Second, cognitive change strategies (e.g., positive appraising of events and looking at the bright side of an event by minimizing its negative effects) were highly efficient in inducing or increasing the intensity of positive emotions. Finally, response modulation strategies (e.g., smiling, laughing, and capitalizing – sharing with others) have also had encouraging outcomes. Response modulation strategies occur after the activation of the emotional response and directly impact the physiological, behavioral, or experiential component of the emotion (for a review, see Quoidbach et al., 2015).

In addition, humor can also be used as a distinct strategy to regulate emotions (Samson and Gross, 2012; Horn et al., 2018; Perchtold et al., 2019), either as a way of distracting oneself from negative emotions (i.e., an attentional deployment strategy; Strick et al., 2009), or as a way to reappraise events (i.e., as a cognitive change strategy; Samson et al., 2014a; Kugler and Kuhbandner, 2015). Therefore, humor can be considered a multifaceted adaptive strategy to regulate emotions, sharing characteristics with different ER strategy categories. In the current paper, however, the use of humor is referred to as a separate strategy meant to attain positive ER goals.

Using various methodologies, cognitive reappraisal (a cognitive change strategy aiming at reinterpreting the meaning of an emotional situation in order to change the subsequent emotion) and expressive suppression (a response modulation strategy aiming at not displaying any emotional response) are the most studied ER strategies in ASD. The use of cognitive reappraisal is linked to long-term beneficial outcomes, whereas the frequent use of expression suppression is linked to long-term detrimental outcomes including higher levels of distress or depression (Gross and John, 2003; Aldao et al., 2010; Schäfer et al., 2017). Individuals with ASD seem to use cognitive reappraisal less spontaneously and with reduced efficacy than typically developing individuals (Samson et al., 2012, 2015b,c). Some studies show a more frequent use of expressive suppression in ASD (Samson et al., 2012, 2015c), while others show similar levels compared to typically developing participants (Samson et al., 2015a,b).

Only a few studies have explored positive emotions in ASD, reporting that the experience and expression of positive emotions in individuals with ASD is attenuated (Dawson et al., 1990; Jaedicke et al., 1994; Hirschler-Guttenberg et al., 2015) and might even be linked to symptom severity (Macari et al., 2018). Parent reports have indicated that children with ASD experience less amusement than their typically developing peers (Samson et al., 2015c). However, adult participants with ASD reported similar levels of positive emotions compared to typically developing participants (Samson et al., 2012). Authors assumed that this unanticipated result was due to the use of emotion-related questions that were context free (and not only limited to social contexts, in which comparatively lower levels of positive emotions could be expected in ASD). To our knowledge, only one study has investigated both the experience of positive emotions and ER in youth with ASD (Samson et al., 2015c). Therefore, any conclusions about the link between these two concepts are still hard to be drawn.

These inconsistent findings on ER strategies and positive emotions may be explained not only by individual differences in the general ASD population, but also by methodological differences between studies including the differences between self and caregivers’ reports (Cai et al., 2018). The difficulty to reliably report the emotional experience and expression of individuals with ASD might partially be related to the reduced emotional coherence (i.e., coordinated changes across emotional response systems: subjective experience, expression, and physiology) found in individuals with ASD in experimental paradigms inducing negative emotions (Costa et al., 2017). Emotional response incoherence in ASD is also observable in the context of positive emotions, or more explicitly, in relation to amusement and laughter toward humorous stimuli (Weiss et al., 2013). Several other studies support the idea of the presence of emotional incoherence in ASD. It has been shown that individuals with ASD tend to have flat affect (Yirmiya et al., 1989) and portray reduced facial expressivity of emotions (Owada et al., 2018), which may at times be perceived as unusual (Grossman et al., 2013; Faso et al., 2014), or as less context-appropriate (Begeer, 2005; Weiss et al., 2013; Costa et al., 2017). Given these rather atypical characteristics, individuals with ASD may benefit from interventions focused on positive ER strategies (e.g., response modulation), helping them enrich their emotional experience (Cai et al., 2018), and engage in and savor positive activities (Carl et al., 2013; Bower, 2015; Taylor et al., 2016).

Interventions incorporating an ER component, such as mindfulness, cognitive behavioral therapy, and dialectic behavioral therapy, have been shown to be beneficial to individuals with ASD, but mostly in young children (Scarpa and Reyes, 2011; Weston et al., 2016; e.g., Conner et al., 2019; Factor et al., 2019; Hartmann et al., 2019; Rispoli et al., 2019). Primarily oriented to reduce negative emotions (e.g., anxiety and anger), these programs implicitly employ strategies linked to attentional deployment and cognitive change (Moyal et al., 2014). Yet, to our knowledge, positive ER tools have not been tested in individuals with ASD.

The goal of the current proof of concept study was to develop a psycho-educational program to explicitly train adaptive positive ER strategies in individuals with ASD and provide the first data testing its practical potential and efficacy. The three-session multimedia program sought to broaden the ER repertoire with a particular focus on positive emotions. Three main adaptive ER strategies were targeted as: attentional deployment, cognitive change, and response modulation. Additionally, the training briefly presented humor as an ER strategy. Our first goal was to test acceptability of the program: Participants’ satisfaction with the program was assessed after each session, and dropouts and aversive events were reported throughout the sessions as indicators of acceptability of the training program. Second, we examined the preliminary effects of the program on the subsequent use of ER strategies: We expected increased self-reported use of the three main strategies post-training and hypothesized that this increase would maintain over time. We have also examined exploratorily the effect of the program on the use of humor as an ER strategy.

## Materials and Methods

### Participants

Thirty French-speaking male participants with ASD took part in the study (*N*_training_=14; *N*_waitlist_=16) without compensation. Groups did not differ significantly in age, socioeconomic status (i.e., the average of parents’ income and education level), participants’ educational background, or enrollment in therapies, nor on parent-reported symptom severity (Social Responsive Scale-2, SRS-2; Constantino and Gruber, 2012) or autistic traits (Autism Spectrum Quotient Short; AQ-short; beside Hoekstra et al., 2011, Bastien (n.d.), unpublished). Participants were either enrolled in school (*N*=18), apprenticeship programs (*N*=8), and university (*N*=2) or were in a transition period after finishing school (*N*=2). See Table 1 for more details.

**Table 1:** Sample characteristics.

	Training Group (*N*=14)	Waitlist group (*N*=16)	Statistics
	**M (SD)**	**M (SD)**	
Age (years)	17.79 (6.52)	18.44 (6.37)	*t*(28) = −0.28, *ns*
SRS-2 (T-score)	74.14 (10.20)	78.31 (7.37)	*t*(28) = −1.30, *ns*
AQ-Short (Total Score)	78.79 (11.64)	82.75 (6.22)	*t*(28) = −1.18, *ns*
Parents’ education level^a^	3.32 (0.61)	3.02 (0.88)	*t*(28) = 1.03, *ns*
Parents’ income level (CHF)^b^	5.38 (1.66)	5.29 (2.13)	*t*(25) = 0.13, *ns*
**Participants’ educational background (*N*)** ^c^			
General/Special	7/7	8/8	*X*^2^(1,30) = 0*, ns*
**Therapy (*N*)** ^d^			
Yes/No	10/4	10/6	*X*^2^(1,30) = 0.27*, ns*
**Assistance during intervention (*N*)**			
Experimenter/Alone	10/4	13/3	*X*^2^(1,30) = 0.40*, ns*

a Answers choices ranged from 1=compulsory education to 4=university.

b Data available for 27 participants (*N*_training_=13; *N*_waitlist_=14). Three parents chose to not answer the question. Answers choices ranged from 1=less than 15000CHF to 10=more than 240000CHF.

c Given the variability of practices across participants and country regions, special education represents here the number of participants who have had one of the following interventions at any time during their education: special education classroom, special education teacher or aide, adapted curriculum or program, reduced number of students in classroom, or inclusive classroom.

d The types of therapy that participants were following at the moment of the intervention include one or more of the following types: occupational therapy, speech therapy, psychological or psychiatric counseling or psychotherapy, or/and psychomotor education.

One participant completed the self-reported forms.

### Procedure

**Figure 1:**
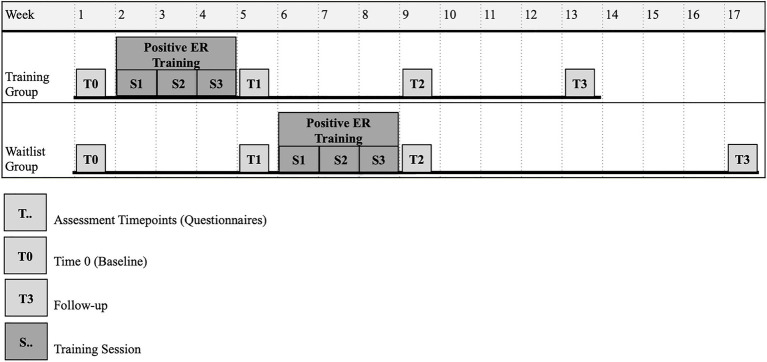
Study timeline of training sessions and questionnaires administered over the weeks.

Participants and, when available, parents were interviewed and participants were screened for inclusion criteria, such as comprehension of verbal instructions, ASD diagnosis established by a qualified healthcare provider, and confirmation of ASD symptomatology (SRS-2 and AQ-short). Twenty-eight participants fell within the clinical range on both scales, the two other participants on one. Using a waitlist control group design, participants were quasi-randomly attributed to either a training group or waitlist group, depending on their time schedule availability. Written informed consent was obtained by participants aged 18 or above and by the parents for participants aged below 18years old or under guardianship. The study was approved by the local ethics committee. The three interactive sessions of 45min each were presented on a computer and administered individually. After each session, participants reported their satisfaction with training and received handouts with examples of exercises that they had tried during training and could also be practiced at home. Participants completed the self-report questionnaires (at four time points; see Figure 1): The training group completed the questionnaires at 7days before training, and at 7, 35, and 63days after training; the waitlist group completed the questionnaires at 35 and 7days before training, and at 7days and 63days after training. As shown in Figure 1, the training group started the three-session intervention the following week after their first self-report questionnaire assessment (right after T0), and the waitlist group started it the following week after the second self-report questionnaire assessment (after T1). The training sessions were completed with experimenters’ assistance only (face-to-face or online) or alone, at home or in our laboratory, depending on each participant’s preference, geographical location, and/or their availabilities. The waitlist group received the intervention after the second self-report assessment (see Figure 1). The study was conducted between 2016 and 2018, before the sanitary restrictions imposed by the coronavirus (COVID-19) pandemic.

### Material

#### Psycho-Educational Training Program

The training program included a child and an adult version that contained appropriate examples for each developmental age range.

##### Content

**Figure 2:**
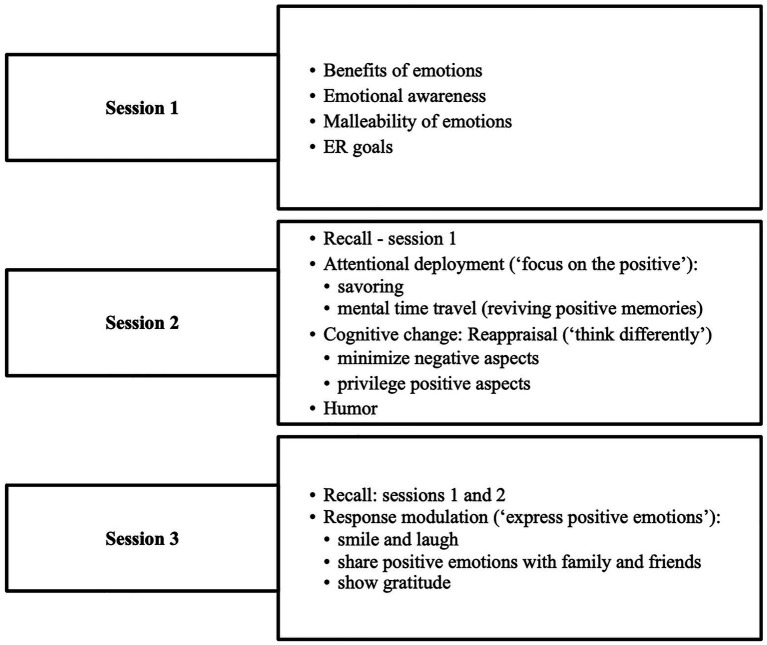
Session content of the positive emotion regulation training program.

During the first session, participants learned about emotional awareness, malleability of emotions, and ER goals, such as trying to regulate emotions that are unpleasant and unhelpful in a particular context. They learned how emotions emerge, what triggers them, and about their functions and benefits. The second session focused on attentional deployment (referred to as “focus on the positive”) and on cognitive change strategies (oriented on cognitive reappraisal – “think differently”), as well as on humor. The third session discussed response modulation strategies (“express positive emotions”). The different techniques used for each strategy are shown in Figure 2.

##### Structure

Each session included theoretical background material, examples, and practical exercises. To facilitate comprehension and render the training interactive, the sessions contained text, images, and animated cartoons created using the online platforms (Moovly - Animation Maker, 2017) and GoAnimate for Schools (Stratton et al., 2014). Examples representing concrete applications of ER in relevant daily life situations (e.g., at school/workplace, with family, or friends) were also shown. Practical exercises included elaborating on participants’ own emotional experiences. For example, they were asked to report situations eliciting positive emotions, or to recall positive memories, and were invited to implement ER strategies retrospectively.

#### Acceptability Measures

After each session, participants were asked to provide feedback about the training program. They evaluated how difficult, interesting, pleasant, and likable the session was. Participants also evaluated the novelty of the exercises, i.e., how often they practice such exercises, as the ones presented in the session, in their daily life. The Likert scale ranged from 1=“not at all” to 5=“very much.” Dropouts and adverse events were recorded.

#### Efficacy Measures of ER Strategy Use

Different self-reported subscales were used to assess the use of the three main *ER Strategies*: the Attentional Deployment subscale (six items, *α*=0.89) of the Attentional deployment/Suppression Questionnaire (e.g., “When I want to feel less negative emotion, I fantasize about doing activities that I really enjoy.”; Barros, 2005); the Cognitive Reappraisal subscale (six items, *α*=0.74) of the Emotion Regulation Questionnaire (e.g., “When I want to feel more positive emotions, I change the way I’m thinking about the situation.”; Gross and John, 2003; Gosling et al., 2018); and the Response Modulation subscale (six items; *α*=0.49) which includes two items from the Berkeley Expressivity Questionnaire (Gross and John, 1997), two from the Emotional Expressiveness Questionnaire (King and Emmons, 1990), and two new items specifically assessing positive response modulation (e.g., “Whenever I feel positive emotions, people can easily see what I am feeling”). Regarding the exploratory hypothesis, *Humor* was assessed with four new items (*α*=0.51; e.g., “When I go through negative or unpleasant events, I try to find something funny about the situation to feel better”). The Likert scale used for the responses ranged from 1=“not at all true” to 5=“definitely true.” Given the low Cronbach’s *α* values at T0 and the higher values obtained at T1 (*α*>0.61), Response Modulation and Humor should be considered with caution.

### Data Analysis

Repeated measure MANOVAs, chi-squared, and *t*-tests were run in IBM SPSS Statistics version 26. Multilevel modeling follow-up analyses and standardized coefficients were run in R software, version 3.6 (R Core Team, 2017), using *lmer4*, *lmerTest,* and *parameters* packages (Bates et al., 2014; Kuznetsova et al., 2017; Lüdecke et al., 2020).

## Results

### Acceptability Indicators

Descriptively, participants rated the training as below scale average on Difficulty (*M*=2.26, *SD*=0.99) and Novelty (M = 2.06, SD = 0.98) and above average for Interest (*M*=3.54, *SD*=0.83), Pleasantness (*M*=3.36, *SD*=0.99), and Likability (*M*=3.46, *SD*=1.02). Repeated measures ANOVAs run for each question showed no effect of session or group. No dropout nor adverse events were observed during the sessions.

### ER Strategy Use

#### Training Group vs. Waitlist Group (T0 and T1)

**Figure 3:**
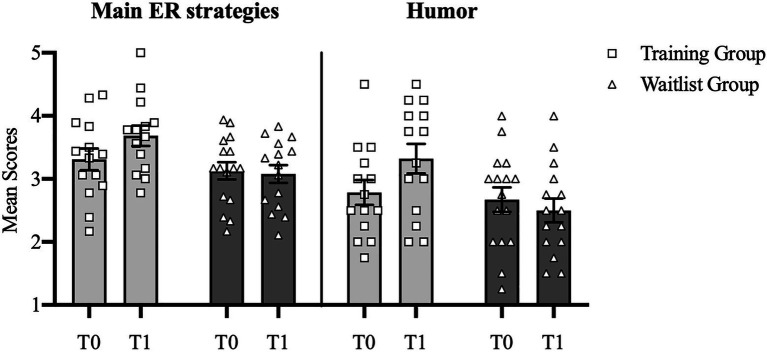
Mean scores of self-reported use of *main ER strategies* and *humor* in training versus waitlist group. Error bars represent standard errors.

First, we expected an interaction effect reflecting increases on the efficacy scores only in the training group. Self-reports for both time points were available for 29 participants (*N*_training_=14; *N*_waitlist_=15). Two separate 2×2×3 MANOVA with two within-subject factors (strategy and time point T0-T1) and one between-subject factor (group) revealed a significant interaction Time point x Group for the main ER strategies (*F*(1,27)=4.31, *p*=0.048, $\eta_{p}^{2}$=0.14) and humor (*F*(1,27)=6.61, *p*=0.016, $\eta_{p}^{2}$=0.20). *Post-hoc* between-group analyses showed that the training group reported a more frequent use of the main ER strategies (*M*_training_=3.68, *SD*=0.60; *M*_waitlist_=3.08, *SD*=0.55, *t*(27)=2.83, *p*=0.009, *d*=1.04) and humor (*M*_training_=3.32, *SD*=0.87; *M*_waitlist_=2.50, *SD*=0.73, *t*(27)=2.76, *p*=0.01, *d*=1.02) than the waitlist group at T1. *Post-hoc* within-group analyses showed that, in the training group, the increase in the use of the three main strategies from T0 to T1 was only marginally significant (*M*_T0_=3.31, *SD*_T0_=0.64, *M*_T1_=3.68, *SD*_T1_=0.60, *t*(13)=−1.89, *p*=0.08, *d*=0.60), whereas the increase in the use of humor was significant (*M*_T0_=2.79, *SD*_T0_=0.74, *M*_T1_=3.32, *SD*_T1_=0.87, *t*(13)=−3.05, *p*=0.009, *d*=0.66). See Figure 3.

#### Entire Sample – Pre- vs. Post-training

**Figure 4:**
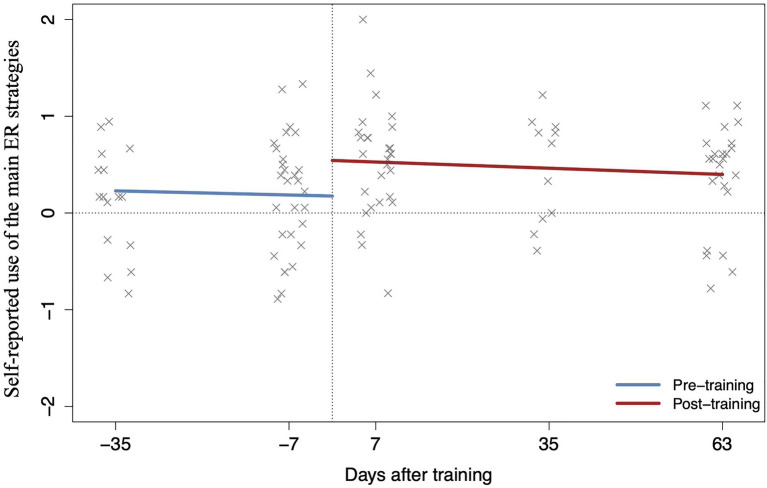
Positive ER training effects over time on use of *main ER strategies* for all the available time points of the entire sample.

**Figure 5:**
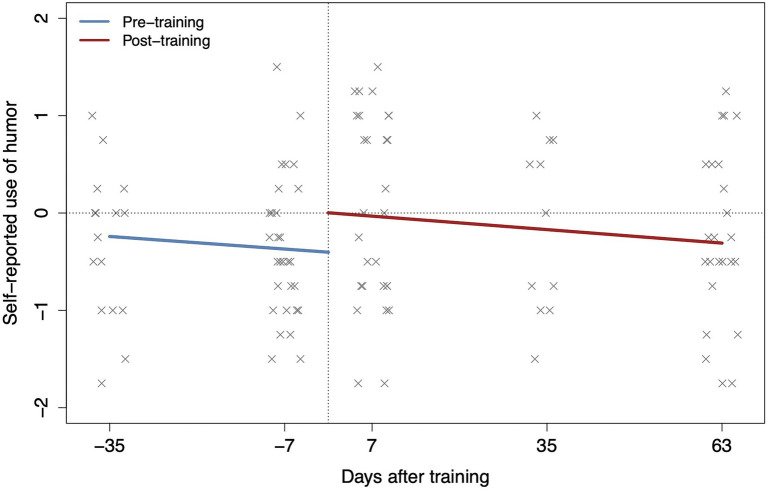
Positive ER training effects over time on use of *humor* for all the available time points of the entire sample.

Next, we expected an increase in the efficacy scores in the entire sample from pre- (combined T0_training_ and T1_waitlist_) to post-training scores (combined T1_training_ and T2_waitlist_). Self-reports for both time points were available for 28 participants (*N*_training_=14; *N*_waitlist_=14). The two separate MANOVAs showed a significant main effect of time point (pre- and post-training) on the main ER strategies (*F*(1,26)=8.51, *p=0*.007, $\eta_{p}^{2}$ = 0.25; Figure 4) and humor (*F*(1,26)=8.21, *p=0*.008, $\eta_{p}^{2}$ = 0.24; Figure 5), indicating an overall increase from pre- (7days before) to post-training (7days after).

#### Multilevel Analyses – The Intervention Effect Over Time

Finally, we hypothesized that intervention effects would persist over time. Based on the overfit evaluation (Akaike information criterion), a multilevel linear model (MLM) including time (i.e., the intervention effect) as a two-level categorical variable (i.e., first level, pre-training, including combined data from 35 and 7days before training; second level, post-training, including combined data from 7, 35, and 63days after training) with a main effect of continuous time (two parallel slopes) was retained. The analyses included all available participants’ time points (*N*=108). The results showed a main effect of the intervention on the use of the main ER strategies (*F*(1,40.65)=10.17, *p=*0.003, *β*_z_=0.65; Figure 4). Comparison analyses indicated a significant increase at 7days (*p*=0.004, *β*_z_=0.59; confirming the previous MANOVAs’ results) and 35days (*p*=0.021, *β*_z_=0.51) after training, and a marginally significant increase 63days later (*p*=0.068, *β*_z_=0.37) compared to 7days before training. A total of 77% of participants (23 out of 30) reported an increase in the use of the main ER strategies post-training. The intervention effect was also significant for humor: *F*(1,78.93)=6.08, *p=*0.015, *β*_z_=0.51 (Figure 5). The only significant increase in the use of humor was found 7days after training (*p*=0.017, *β*_z_=0.44). Both analyses showed a main effect of continuous time at pre- and post-intervention on the main ER strategies: *F*(1,48.86)=5.15, *p=*0.003, *β*_z_=−0.12 and humor: *F*(1,80.15)=4.01, *p=*0.049, *β*_z_=−0.19, which could be due to differences in the sample size (35 vs. 7days before intervention). The results did not change when age was introduced as a covariate.

## Discussion

### Main Findings

#### Acceptability

Overall, the program has received high evaluations and was proven to be adequate in terms of acceptability. Participants mainly rated the program as easy to understand, interesting, pleasant, and likable. On average, participants indicated that they do not usually use these strategies in their daily lives. The novelty of the information presented during the training program might have contributed to the interest that participants have reported throughout the sessions and might explain the full adherence to the program. As no dropouts or adverse effects were observed during sessions, this brief program has proven to have exemplary attrition and safety rates. Although these observations could probably be explained by the brevity of the intervention, they nonetheless encourage further use of such training programs with individuals with ASD.

#### ER Strategy Use

The current study revealed promising effects of using this psycho-educational training program on positive ER in individuals with ASD. As expected, participants used the three main ER strategy types (attentional deployment, cognitive change, and response modulation) more frequently post-training. This result was confirmed in a larger sample with both groups pooled together when we measured the changes immediately before and after the intervention. Importantly, despite the brevity of the program, the observed effects on the use of the main ER strategies were maintained until at least 7weeks later. Our findings indicate that individuals with ASD are able to learn new strategies promoting positive emotions. This suggests that they can benefit from interventions targeting the improvement of positive ER skills.

An exploratory analysis also revealed an increase in the self-reported use of humor as an adaptive ER strategy. While this may be interesting, this result should be considered with caution given the unstable Cronbach’s alpha of the humor scale which shows low reliability at T0 and high reliability at T1. The change in scale reliability (T0 vs. T1) could be explained by various factors often observed in educational research: the sample homogeneity at T0, the intervention effects (changes between measurements), or the measurement process (activation of new insights about the question; e.g., Taber, 2018). Future research should assess the use of humor as an ER strategy using more reliable scales.

### Limitations and Future Perspectives

First, several methodological limitations are worthy of note. One limitation is the relatively small sample size. Yet, although for a proof of concept study, a small sample is appropriate, the intervention should be more rigorously tested in larger samples in order to see whether these initial findings generalize as well as in order to increase the statistical power in the analyses. However, importantly, the effect sizes of the cross-sectional analyses, as well as the standardized coefficients of the MLM analyses (*β*_z_), indicate medium to strong effects of the intervention on the use of the main positive ER strategies and humor. Another methodological limitation is the waitlist control group design. Certain researchers recommend the use of “stronger” designs (e.g., active control groups) which can limit the potential participants’ expectation biases and control for non-specific intervention effects (see Kinser and Robins, 2013). However, a waitlist control group design has important advantages: It is more cost-effective, especially in the first phases of testing a new intervention program, and it is more suitable for ethical considerations, as all participants can eventually benefit from the intervention (see Moore and Ledbetter, 2020). Finally, although the researchers ensured that the subscales contain items easy to understand at all ages, these measures, taken together, should be further validated in young samples and in samples with ASD. For instance, only the Cognitive Reappraisal scale has already been validated in a typically developing child and adolescent population within an age range from 8 to 16years old (Gosling et al., 2018), whereas the Berkeley Expressivity Questionnaire and the Emotional Expressiveness Questionnaire have been used with children of 14 years of age and older (e.g., Doostian et al., 2015; Akkuș Çutuk, 2021). Overall, the present participants did not report difficulties in understanding the content of the self-reported measures, but they did sometimes report a certain lack of motivation toward the completion of the questionnaire and its repetitiveness over the four time points.

A second limitation is that ASD diagnosis was not confirmed using the gold standards (e.g., ADOS-2; Lord et al., 2012) and neither cognitive skills nor adaptive functioning were objectively assessed. Nonetheless, we believe that our program was proven to be suitable for verbally fluent individuals with ASD, whereas assistive technologies (e.g., smartwatch; Torrado et al., 2017) may be more appropriate for minimally verbal individuals with ASD.

Third, gender and age differences may be important. Extending this research to females could improve understanding of ER in individuals with ASD (Trubanova et al., 2014; Cai et al., 2018). Not only is ASD under-identified in females (Rynkiewicz et al., 2016), it has also been suggested that emotion dysregulation could represent a key factor contributing to the unrepresentative ASD symptomatology in females (Trubanova et al., 2014). Differences between males and females in the use of ER strategies have also been found in typically developing populations (Cai et al., 2018). To date, only one study has examined this topic and found that females with ASD had slightly more emotion regulation difficulties compared to males with ASD: They were more prone to experience dysphoria and faced more impairments related to high emotional intensity (Trubanova Wieckowski et al., 2020). Future studies should explore the efficacy of such interventions in female individuals with ASD. Furthermore, as ER patterns change with age (Samson et al., 2012; Cai et al., 2018), identifying the most frequently used ER strategies and the specific ER impairments at each developmental stage could help provide a more individualized use of interventions. Although the literature suggests that the adaptive ER strategies are the same across development (Aldao et al., 2010; Schäfer et al., 2017), we would expect certain developmental differences in the frequency of use and efficacy of these strategies (Garnefski et al., 2002).

Also, while the data indicate a more frequent use of ER strategies after the training, it remains unknown how well people successfully completed the ER phases in their daily life (e.g., strategy selection and implementation; Gross, 2015). Previous research has shown that the flexible use of ER strategies is worthy of further investigation, as it might be a key factor contributing to an adaptive ER and influence ER efficacy (Aldao et al., 2015; Ford et al., 2019; Kobylińska and Kusev, 2019). Multi-method approaches using performance tasks (Samson et al., 2015b), virtual reality (e.g., Ip et al., 2018), and physiological measures as well as daily diaries or ecological momentary assessments could be of help to test the flexible use of various ER strategies across different contexts in individuals with ASD (Cai et al., 2018).

While this study shows high acceptability rates and promising first effects on the increased use of adaptive positive ER strategies post-training, future research should examine the impact on proximal and distal outcome measures (e.g., emotion experience, wellbeing, and socio-emotional functioning) with appropriate scales to capture changes (Samson et al., 2012; Vermeulen, 2014) in larger efficacy studies. A higher and more improved impact of the intervention is expected to occur after upgrading the current version into an intensive program with an increased number of sessions, while considering the likelihood that the dropout rates might increase. A closely monitored practice between sessions could also be more impactful and help measure adherence to the program, in addition to the acceptability measures that have already shown highly satisfactory results. Finally, adapting the program into a caregiver-mediated intervention could also contribute to obtaining better outcomes, especially in younger participants (Rispoli et al., 2019).

### Conclusion

This first version of our positive ER training has been shown to be participant-friendly and appropriated for individuals with ASD, and indicated promising preliminary effects on the participants’ self-reported use of adaptive ER strategies after the training program. This new program could be a valuable tool for practitioners and clinicians to train ER skills. It could also supplement the need for online intervention tools, not only in times when sanitary restrictions require the implementation of remote sessions to support patients with developmental disorders (e.g., COVID-19; Grumi et al., 2020), but also to facilitate, in general, the delivery of care beyond it (Jeste et al., 2020). The findings of the current proof of concept warrant future research on this topic, which could shed more light on the generalizability of the outcomes and the role that positive ER plays in the onset, manifestation, and development of challenging behaviors in individuals with ASD. Importantly, it will be necessary to examine the impact of the training on positive emotions in individuals with ASD. Adaptive emotion regulation skills as well as positive emotions may play an important role in social and adaptive functioning including school inclusion, education, and transition into a professional life as well as wellbeing in individuals with ASD.

## Data Availability Statement

The raw data supporting the conclusions of this article will be made available by the authors, without undue reservation.

## Ethics Statement

The study was performed in accordance with the Declaration of Helsinki and was approved by the Swiss Ethical Committee Board of Geneva (Date 01.03.2016/No. PB_2016-00750/15-242). Written informed consent to participate in this study was provided by the participants aged 18 or above and by parents for participants aged below 18 years or under guardianship.

## Author Contributions

AS developed the design of study, acquired funding, and supervised the study. DS contributed to the study conception and design. AZ administered the project, prepared the research material, contributed to the training program content, organized the database, performed the statistical analysis, and wrote the first draft of the manuscript. Data collection was performed by AZ, KN-K, and NB. All authors reviewed and commented the previous versions of the manuscript, and read and approved the final manuscript.

## Funding

This research was supported by the Swiss National Science Foundation (SNSF: PA00P1–154937 and PP00P1_176722 for AS, SNSF: P2NEP1–178584 for DD) and the Réseau Fribourgeois de Santé Mentale (RFSM), Fribourg, Switzerland (for KN-K).

## Conflict of Interest

The authors declare that the research was conducted in the absence of any commercial or financial relationships that could be construed as a potential conflict of interest.

## Publisher’s Note

All claims expressed in this article are solely those of the authors and do not necessarily represent those of their affiliated organizations, or those of the publisher, the editors and the reviewers. Any product that may be evaluated in this article, or claim that may be made by its manufacturer, is not guaranteed or endorsed by the publisher.
